# Think globally and solve locally: secondary memory-based network learning for automated multi-species function prediction

**DOI:** 10.1186/2047-217X-3-5

**Published:** 2014-04-23

**Authors:** Marco Mesiti, Matteo Re, Giorgio Valentini

**Affiliations:** 1 AnacletoLab - Department of Computer Science, University of Milano, Via Comelico 39/41, 20135 Milano, Italy

**Keywords:** Biomolecular networks, Big data analysis, Network-based learning

## Abstract

**Background:**

Network-based learning algorithms for automated function prediction (AFP) are negatively affected by the limited coverage of experimental data and limited *a priori* known functional annotations. As a consequence their application to model organisms is often restricted to well characterized biological processes and pathways, and their effectiveness with poorly annotated species is relatively limited. A possible solution to this problem might consist in the construction of big networks including multiple species, but this in turn poses challenging computational problems, due to the scalability limitations of existing algorithms and the main memory requirements induced by the construction of big networks. Distributed computation or the usage of big computers could in principle respond to these issues, but raises further algorithmic problems and require resources not satisfiable with simple off-the-shelf computers.

**Results:**

We propose a novel framework for scalable network-based learning of multi-species protein functions based on both a local implementation of existing algorithms and the adoption of innovative technologies: we solve “locally” the AFP problem, by designing “vertex-centric” implementations of network-based algorithms, but we do not give up thinking “globally” by exploiting the overall topology of the network. This is made possible by the adoption of secondary memory-based technologies that allow the efficient use of the large memory available on disks, thus overcoming the main memory limitations of modern off-the-shelf computers. This approach has been applied to the analysis of a large multi-species network including more than 300 species of bacteria and to a network with more than 200,000 proteins belonging to 13 Eukaryotic species. To our knowledge this is the first work where secondary-memory based network analysis has been applied to multi-species function prediction using biological networks with hundreds of thousands of proteins.

**Conclusions:**

The combination of these algorithmic and technological approaches makes feasible the analysis of large multi-species networks using ordinary computers with limited speed and primary memory, and in perspective could enable the analysis of huge networks (e.g. the whole proteomes available in SwissProt), using well-equipped stand-alone machines.

## Background

In recent years many efforts have been devoted to build automated tools for large scale automated function prediction of proteins (AFP) exploiting the knowledge generated by high throughput biotechnologies [1,2]. As highlighted by a recent international challenge for the critical assessment of automated function prediction [3], scalability and heterogeneity of the available data represent two of the main challenges posed by AFP. Indeed on the one hand no single experimental method can fully characterize the multiplicity of the protein functions, and on the other hand the huge amount of data to be processed poses serious computational problems. The complexity of the problem is furthermore exacerbated by the different level of the functional annotation coverage in different organisms, thus making very difficult the effective transfer of the available functional knowledge from one organism to another.

Computational automated function prediction approaches can be useful for the integration of diverse types of data coming from multiple, often unrelated, proteomic and genomic pipelines. A recent example is represented by the Integrative multi-species prediction (IMP) web server [4] which integrates prior knowledge and data collections from multiple organisms for the generation of novel functional working hypotheses used in experimental follow-up. Despite its undoubted usefulness, IMP actually covers only seven model organisms, preventing its application to the prediction of the functions of proteins belonging to the proteomes of poorly annotated organisms.

Another popular approach for gene functional annotation transfer between species relies on the availability of a collection of orthology relationships across interspecies proteins, and on the usage of an evolutionary relationships network as a suitable medium for transferring functional annotations to the proteins of poorly annotated organisms [5]. Even if orthology is an evolutionary concept, rather than a functional one, it can be used to link functionally equivalent genes across genomes and enables the functional inference of unknown proteins using one or more functionally characterized orthologs in other species [6,7].

As noticed in [4], the accuracy of machine-learning algorithms for AFP tasks is negatively affected by the sparse coverage of experimental data and by the limited availability of prior functional knowledge. Consequently, these methods are often applied only to biological processes and pathways that are already well characterized for an organism. The construction of large scale multi species networks can be a solution to this problem. Following this approach, network based learning algorithms might benefit of the availability of a priori functional knowledge coming from well annotated species to effectively perform a functional transfer to the proteins of poorly annotated organisms.

Unfortunately this solution is only apparently simple, since the application of classical graph-based algorithms such as the ones based on random walks [8] or label propagation methods [9,10] are often unfeasible with large multi-species networks, especially when only single off-the-shelf machines are available. These approaches, indeed, usually rely on an in-memory adjacency matrix representation of the graph network, scale poorly with the size of the graph [11], and may have time complexity that becomes quickly prohibitive. Performance optimization is usually realized by adopting an adjacency-list representation of the graph to take its sparsity into account, or by using parallel strategies for matrix multiplication [12]. However, when the size of the graph becomes so high that is not possible to maintain it entirely in primary memory, either approaches based on parallel distributed computation [13-15], or secondary memory-based computation [16-18] can be considered. With distributed computation techniques, the graph is spread on different machines and the results are finally collected. However, as outlined in [16], a key issue of these approaches is the need to identify a cut of the graph in order to minimize the communication overhead among machines and their synchronization activities. With secondary memory-based computation, the graph is stored on the disk of a single machine and only limited portions of the graph are loaded in primary memory for computation. In this way, it is possible to overcome the lack of enough primary memory. The use of smart strategies for caching the portions of graph needed for computation [19], the minimization of the number of accesses to secondary memory [20], and the usage of compressed data structures for maintaining the graph in primary memory [21] are the main challenges for making the management of large graph networks in off-the-shelf machines comparable to distributed approaches.

In this work we propose a novel framework for scalable semi-supervised network-based learning of multi-species protein functions: on the one hand we adopt a “local learning strategy” to implement classical graph-based algorithms for protein function prediction, and on the other hand we apply secondary memory-based technologies to exploit the large disks available in ordinary off-the-shelf computers. The combination of these algorithmic and technological approaches makes feasible the analysis of large multi-species networks in ordinary computers with limited speed and primary memory and in perspective could enable the analysis of huge networks (e.g. the whole proteomes available in SwissProt), using well-equipped stand-alone machines.

Only very recently a paper has been devoted to the application of graph database technologies in bioinformatics [22], and to our knowledge this is the first work where secondary-memory based network analysis has been applied to multi-species function prediction using big biological networks with hundreds of thousands of proteins.

This paper is organized as follows. In the next section we introduce our proposed approach based on the local implementation of network-based algorithms and secondary memory-based computation for the multi-species AFP problem. In particular we discuss the characteristics of *Neo4j*, a database technology for graph querying and processing, and *GraphChi*, a disk-based system for graph processing. Then, we show their application to a multi-species network involving proteins of about 300 bacteria species, and to a network including 13 species of Eukaryotes with more than 200.000 proteins, using off-the-shelf notebook and desktop computers.

## Methods

Our approach to big-graph analysis for AFP leverages on both a novel computational model for network analysis and on novel technologies for fast and efficient secondary memory-based computation. More precisely we adopt at the same time two strategies for scalable network-based learning of protein function: 

1. *Local Implementation* of network-based algorithms. To solve the overall AFP problem we adopt a local learning strategy, according to a “vertex-centric” computational model.

2. *Secondary memory-based computation*. We exploit novel technologies for fast and efficient secondary-memory access: the overall graph is maintained on disk and only small parts of it are loaded each time into primary memory.

It is worth noting that we do not propose novel algorithms, but simply their “local implementation”, according to a vertex-centric programming model, necessary for secondary memory-based computation [14]. Indeed the strength of the proposed approach consists precisely in coupling a “local” vertex-centric implementation of network-based algorithms with technologies based on secondary memory, to make efficient the local access to graphs stored on disk, thus also allowing the processing of big biological networks when limited RAM memory is available.

### Local implementation of network-based algorithms

The most effective network-based algorithms for AFP learn by exploiting the overall topology of the networks [23-25], and their implementation usually requires to process in primary memory a large part or the overall underlying graph. The main drawback of this implementation is that big networks cannot be entirely loaded into primary memory using off-the-shelf machines.

We aim at providing local implementations of “global” network algorithms by iteratively processing only one vertex and its incident edges at a time. In other words we do not reject to think “globally” by exploiting the overall topology of the network, but at the same time we solve “locally” by designing implementations of these algorithms through a vertex-centric programming model [14,26].

As an example, we consider the local implementation of the “vanilla” random walk (*RW*) algorithm [8], a popular network-based method just successfully applied to AFP [24]. It is worth noting that the *RW* algorithm is “global”, in the sense that it may exploit the global topology of the graph, but it is also intrinsically local, since at each step each vertex can be processed considering only its direct neighbours. From this standpoint its local implementation is straightforward, since it is sufficient to iteratively process each vertex, its edges and its directly connected vertices to obtain a “vertex-centric” implementation of the algorithm. Other algorithms that can process the adjacency matrix of a graph row by row (e.g., label propagation algorithms [9]) can be easily implemented according to a vertex-centric programming model and can benefit from disk-based approaches. More in general the proposed approach can be extended to any other network-based method for which a local implementation can be provided.

#### Basic notation

Having a graph *G*=<*V*,*E*>, representing a functional network, where the vertices *V* correspond to proteins, and edges *E* to functional relationships between proteins, we indicate proteins with integers, i.e. *V*={1,2,…,*n*}, where *n*=|*V*| is the number of vertices/proteins, and edges (*i*,*j*)∈*E* represent functional relationships between vertices *i*,*j*∈*V*. The weights $w_{\text{ij}}\in \mathbb{R}$ associated with edges (*i*,*j*) represent the “strength” of their functional relationships and are elements of the symmetric weight matrix ***W***. *C*⊂*V* indicates the proteins belonging to a functional class *c* (e.g., a specific Gene Ontology (GO) term [27]).

#### Local implementation of random walks

*Random walk* (*RW*) algorithms [8] explore and exploit the topology of the functional network, starting and walking around from a subset *C*⊂*V* of nodes belonging to a specific class *c* by using a transition probability matrix ***Q*** = ***D***^−1^ ***W***, where ***D*** is a diagonal matrix with diagonal elements $d_{\text{ii}}=\underset{j}{\sum }w_{\text{ij}}$. The elements *q*_*i* *j*_ of ***Q*** represent the probability of a random step from *i* to *j*.

The probability to start the walk can be set to *p*^*o*^=1/|*C*| for the nodes *i*∈*C* and to *p*^*o*^=0 for the proteins *i*∈*V*∖*C*. If ***p***^*t*^ represents the probability vector of finding a “random walker” at step *t* in the nodes *i*∈*V* (that is, $p_{i}^{t}$ represents the probability for a random walk of reaching node *i* at step *t*), then the probability at step *t*+1 is:

(1) $$p^{t+1}=Q^{T}p^{t}$$

and the update (1) is iterated until convergence or until a finite number of steps is reached.

From a “vertex-centric” standpoint the update rule (1) of the *RW* algorithm becomes:

(2) $$p_{i}^{t+1}=Q_{i}\cdot p^{t}$$

where *p*_*i*_ is the probability of the *i*^*t* *h*^ node, and *Q*_*i*_ represents the *i*^*t* *h*^ column of the probability transition matrix ***Q***. By recalling that ***W*** represents the original weighted adjacency matrix of the graph and *W*_*i*_ its *i*^*t* *h*^ column, from (2) we obtain:

(3) $$p_{i}^{t+1}=D^{-1}\cdot W_{i}\cdot p^{t}=\overset{n}{\underset{j=1}{\sum }}d_{\text{jj}}^{-1}\;w_{\text{ji}}\;p_{j}^{t}$$

Equation (3) is the update rule of the random walk resolved at the *i*^*t* *h*^ node of the graph, and can be viewed as a “local” version of (1): by updating all the nodes *i* of the graph, 1≤*i*≤*n*, we update the probability vector ***p***^*t*+1^ exactly in the same way of (1). To compute (3) we need the following information: 

1. $d_{\text{jj}}^{-1}\;=\;\frac{1}{\underset{i}{\sum }w_{\text{ji}}}\;$ (i.e., the inverse of the sum of weights of the edges coming from *j*)

2. *w*_*j* *i*_,1≤*j*≤*n* (i.e., the weights of the inedges of *i*)

3. $p_{j}^{t},1\leq j\leq n$ (i.e., the probabilities of node *j* at the previous step).

We can observe the following facts: 

a) If the graph is undirected (and this is the case for the AFP problem), the weights of incoming and outcoming edges are the same, that is ∀*i*,∀*jw*_*i* *j*_=*w*_*j* *i*_. This implies that only the list of edge weights outcoming from *i*: *L*(*i*)={*w*_*i* *j*_|*w*_*i* *j*_>0} should be stored. This in turn implies that in sparse graphs the spatial (and temporal) complexity at each node is sublinear, and (3) can be expressed as:

(4) $$p_{i}^{t+1}=\underset{j\in N(i)}{\sum }d_{\text{jj}}^{-1}\;w_{\text{ji}}\;p_{j}^{t}$$

where *N*(*i*)={*j*|*j*∈*V*∧(*i*,*j*)∈*E*} are the neighborhood vertices of *i*.

b) We need to store $p_{j}^{t},$ and $p_{j}^{t+1}$, 1≤*j*≤*n*, that is the probabilities at the current and previous step. Once a step is completed, the current probabilities ($p_{j}^{t+1}$) can be used as starting probabilities for the next iteration.

c) We can store $d_{\text{jj}}^{-1},1\leq j\leq n$, as a value associated to each node *j*. It could be computed at each node *j* as a pre-processing step: $d_{\text{jj}}^{-1}=\frac{1}{\underset{i}{\sum }w_{\text{ji}}}$.

d) The algorithm iterates for a predefined number of steps or until convergence.

e) It is easy to see from (3) that the complexity of each iteration of the algorithm is $O(n^{2})$, but with sparse graphs, i.e. when ∀*i*,|{(*j*,*i*)|*w*_*j* *i*_>0}|<<*n*, the complexity is O(n).

### Secondary memory-based computation

To be actually applicable to real-world big networks, the local implementations of the algorithm described in Section “Local implementation of network-based algorithms” require specific technologies for an efficient access to the secondary memory: indeed we need to efficiently load small parts of a graph, update them in primary memory and finally store them back to disk.

To this end we experimented with two different secondary memory-based technologies. The first one is based on graph DB technologies [28], and the second one on efficient technologies for disk-based processing of graphs.

#### Neo4j: a DB technology for graph querying and processing

*Neo4j*[17] is a data management system written in Java based on the graph data model. Nodes, relationships and their properties are first class citizen in the model and their storage on disk is optimized by the adoption of specific data structures for graph networks. The *Neo4j* Kernel is a fast graph engine with the main characteristics expected by a DBMS, like recovery, management of transactions and indexing structures. *Neo4j* can be used both as an embedded database within a Java application and as a standalone server with an extensive REST interface for easy integration with Web applications. A declarative query language, named *cypher*, for the specification of SQL-style queries is provided.

Internally, *Neo4j* stores graph data spread across a number of files. Each store file contains the data for a specific part of the graph (e.g. nodes, relationships, properties) and their overall organization, which entails the separation of graph structure from property data, allows the efficient traversal of the graph and the generation of query answers. Both nodes, relationships and properties have a fixed size representation (e.g. nodes have a fixed dimension of 9 bytes), and relationships are implemented using doubly linked lists on disk in order to render efficient their traversal. The fixed-size representation of nodes, relationships and properties has the advantage that identifiers should not be stored (corresponds to the file offset) and that their retrieval by means of their identifiers can be done in constant time.

Since this information is stored in secondary memory, its access is made efficient through the use of caching techniques. At file system level, each store file is divided in equally sized regions and these regions are cached. The cache holds a fixed number of regions for each file, and regions are replaced relying on a least frequently used (LFU)-like policy. On top of this mechanism, a more specific node/relationship cache has been implemented that is optimized for traversal (for example, relationships of a node are organized relying on their type and their direction).

**Figure 1:**
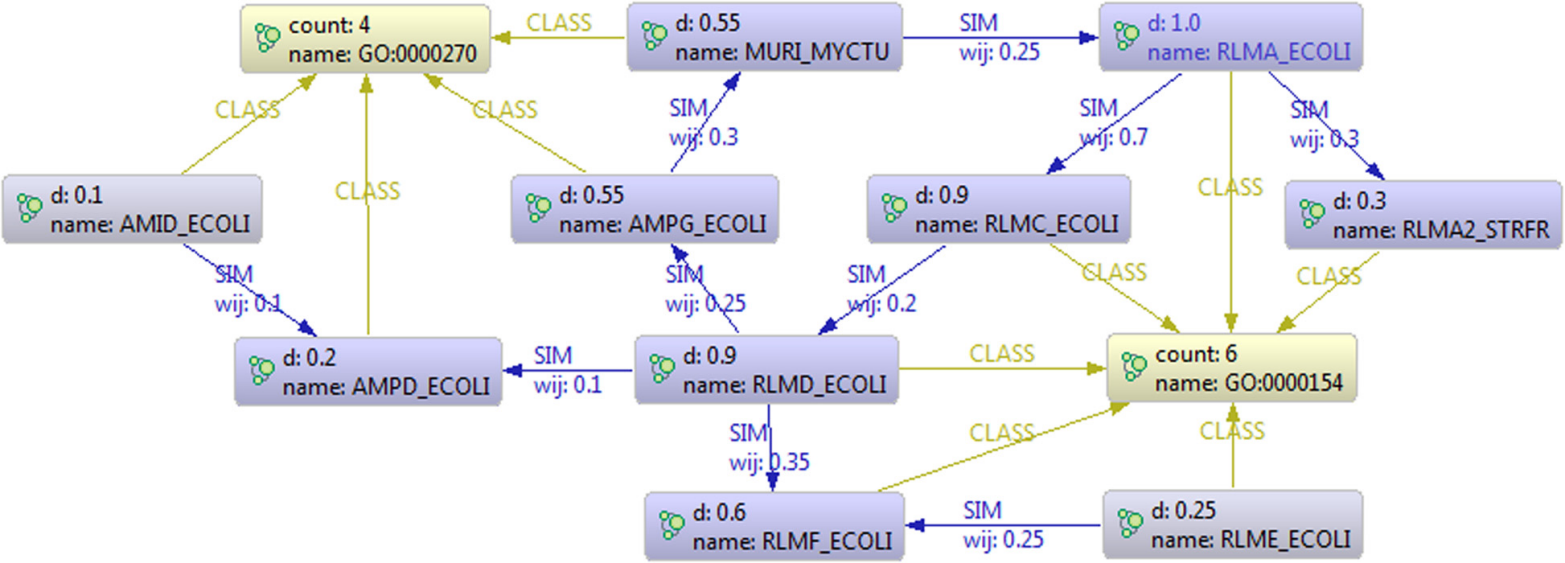
**A sample *Neo4j *net.** A graphical representation of a sample *Neo4j* net.

In *Neo4j* the functional network *G* used for AFP has been implemented as follows. Each node representing a protein *i* is associated with the properties name, d (i.e. $1/\underset{j}{\sum }w_{\text{ij}}$), p1 and p2 (i.e. the probability of the protein at the previous and current step). Moreover, between two proteins *i* and *j* a relationship of type SIM is specified with a property *w*_*i* *j*_ containing the strength of their functional relationship. The graph has been enhanced with nodes representing the functional classes (with name and count properties, i.e. the name of the class and the number of proteins belonging to the class) and relationships of type CLASS, that represent the classes to which a protein belongs to. Figure 1 reports a simple example of the graph with 10 bacteria proteins and two GO terms with their relationships. For the sake of simplicity, the values of p1 and p2 are not reported.

**Figure 2:**
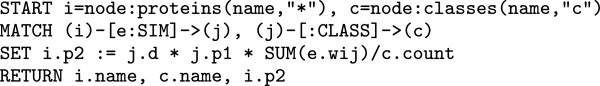
***Neo4j* Implementation of 1-step *RW* algorithm in cypher.** The notation (i)-[e:rtype]->(j) is used to represent a relationship *e* of type *rtype* between nodes *i* and *j*. The dot-notation is used to access a single property of a node/edge.

Even if the *RW* algorithm described in Section “Local implementation of network-based algorithms” has been implemented in Java with the embedded version of Neo4j, it can be easily expressed through the cypher language. This declarative query language allows the expression of the core definition of the “vanilla” *RW* with a single simple statement (Figure 2). More precisely, starting from a generic protein *i* and a function class named *c*, the cypher implementation identifies the proteins *j* for which a relationship of type SIM exists with *i* and such that *j* is of class *c*. Then, the probability *i*.p2 (at time *t*+1) is obtained by using the value *j*.*d* and *j*.p1 (the probability computed at time *t*). Finally the statement returns the name of protein *i*, the name of the class *c*, and the computed probability *i*.p2 (Figure 2).

#### GraphChi: a disk-based system for graph processing

*GraphChi* is a disk-based system for the analysis of big graphs on single off-the-shelf computers [16]. Differently from *Neo4j*, *GraphChi* has not been conceived for querying large graph-structured databases, but for efficiently processing graphs stored in secondary memory. To this end it implements specialized data structures to efficiently break large graphs into small parts that can be quickly loaded into primary memory, and provides efficient disk I/O operations to reduce the number of non sequential accesses to disk. Moreover, it offers an asynchronous model of computation that directly supports the vertex-centric programming model.

**Figure 3:**
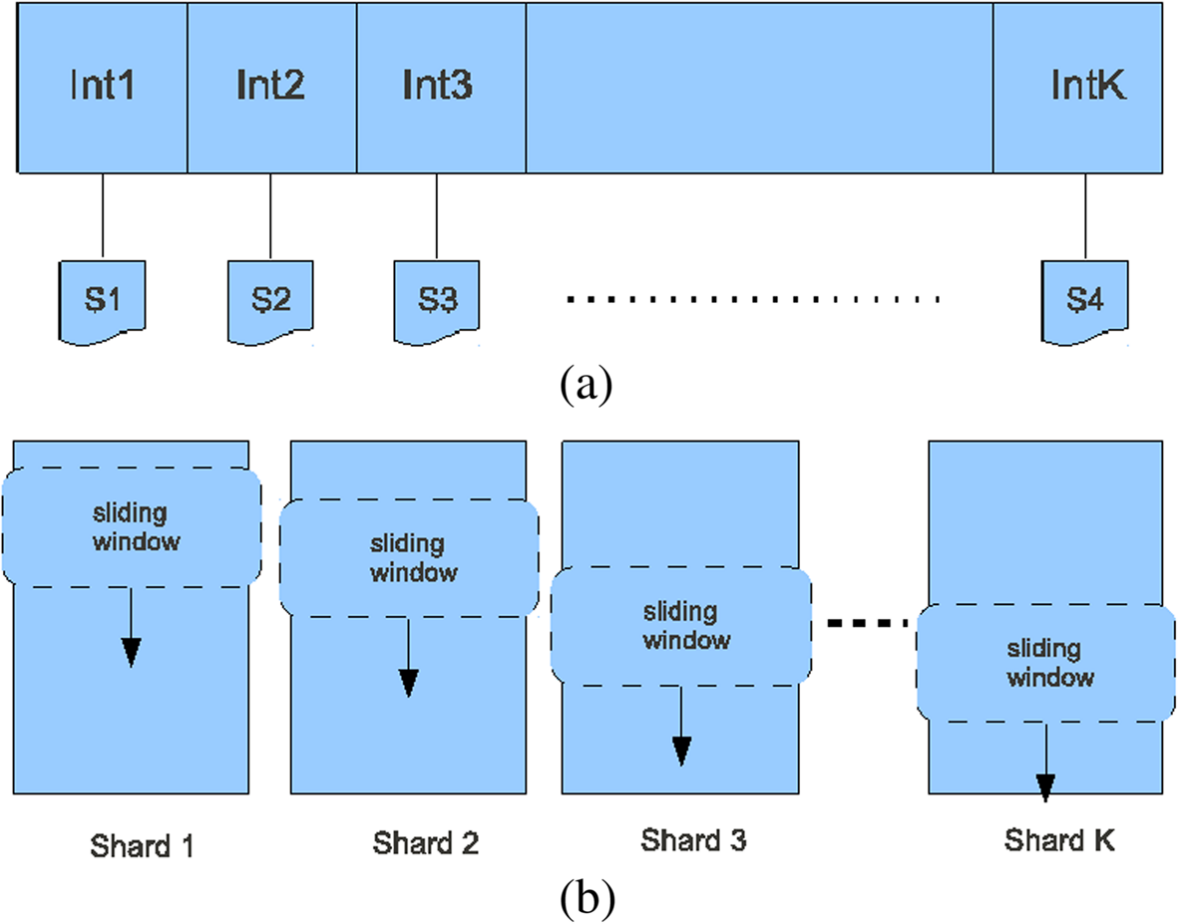
**Efficient disk access with *GraphChi. *(a)** Shards: *Int*1,…*IntK* refer to the *K* intervals in which the vertices are split, while *S*1,…*SK* to the corresponding shards **(b)** Parallel Sliding Windows.

*GraphChi* requires enough primary memory to contain the edges and their associated values of only a relatively small subset of vertices at a time, while the rest of the graph is efficiently stored on disk. More precisely, the vertices of the graph are split in *K* intervals, and each interval is associated to a *shard* which stores all the inedges for the vertices in the interval itself (Figure 3a). Note that the inedges are sorted by their source vertex. The dimensions of the intervals are chosen in such a way that the corresponding shards can be entirely loaded into primary memory: hence all the inedges are available for the vertices in the interval. Moreover, the outedges can be efficiently loaded requiring at most *K* non sequential disk-reads, through the mechanism of the *Parallel Sliding Windows (PSW)*: by exploiting the ordering of the edges with respect to the source vertices, when PSW moves from an interval to the next, it “slides” a window over each of the shards (Figure 3b).

Schematically, the execution flow of *GraphChi* can be summarized in an iterative cycle, repeated across each interval of vertices: 

1. *Read*: select an interval and load in primary memory its inedges stored in the associated shard (the “memory shard”). Through at most *K* non sequential reads load its outedges.

2. *Execute*: perform a parallel update of vertices and edges of the memory shard through multi-thread asynchronous computation in primary memory.

3. *Write*: The updated vertices and edges are written back to disk.

Note that the mechanism of *Parallel Sliding Windows* requires at most *K*^2^ non sequential reads/writes on disk for a full visit of the entire graph (*K* reads/writes for each interval), thus resulting in a very efficient management of primary and secondary memory [16].

**Figure 4:**
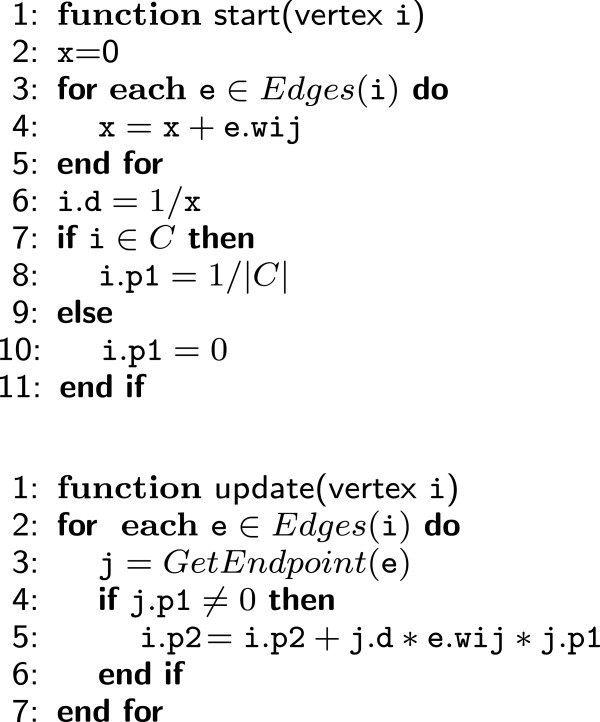
**Pseudocode of the *GraphChi *** **vertex-centric implementation of the 1-step *RW *** **algorithm.**

The *GraphChi* implementation of the *RW* algorithm requires a data structure for representing a vertex containing the same properties specified for the *Neo4J* implementation (namely, d, p1 and p2 – Section “Neo4j: a DB technology for graph querying and processing”). Moreover, a weight is associated with each edge e (referred to as *e*.*w*_*i* *j*_). Figure 4 reports the pseudo-code of the 1-step RW vertex-centric implementation, including the *start* and the *update* functions, that specify the actions to perform on a vertex i during the first and the succeeding update iterations. In the start function each vertex is initialized with the value of d and the initial probability p1. In the update function the probability of the 1-step RW algorithm is determined by simply applying eq. 4. By means of the *GraphChi* execution engine, these functions are automatically applied to all the vertices of the graph, according to a multi-thread and asynchronous mode of computation. This implementation can be easily extended to an arbitrary number of steps by modifying the *update* function in order to read previous probabilities from p1 during the odd iterations and from p2 during the even iterations (and writing the current probability in the other variable).

The C++ implementation of this algorithm in *GraphChi* entails to keep in main memory a global vector containing a copy of the data structures for each vertex *v*∈*V*. Indeed, during the execution of the *update* function, it is only possible to access the identifier of the neighbour vertex j contained in the data structure representing the edge *e*, but not its associated probability *j*.*p*1 and *j*.*d* values, necessary for the computation of the probability *i*.*p*2 (Figure 4). Therefore, the global vector in main memory is used just to access this information. We remark that this solution makes our implementation even faster and feasible in our AFP context, since the number of vertices is by far smaller than the number of edges, and thus there is no risk of running out of memory also with off-the-shelf computers, even for very large biological networks involving hundreds of thousands or even millions of proteins.

## Analyses

We applied our methods based on the local implementation of network-based algorithms and secondary memory-based computation to the multi-species protein function prediction in bacteria and eukarya. In the remainder of the section we summarize the experimental set-up and the characteristics of the data, and then we compare the empirical computational time required by secondary and primary memory-based implementations of network based algorithms for AFP.

### Data description and experimental set-up

We applied our methods to two multi-species networks of proteins: the first one (*Bacteria-net*, Section “Bacteria-net”) accounts 301 species of bacteria, and the second one (*Eukarya-net*, Section “Eukarya-net”) includes the proteomes of 13 Eukaryotic species.

#### Bacteria-net

We constructed a multi-species bacteria network (*Bacteria-net*), using the proteins proposed as part of a large scale experiment for a recent international challenge aimed at the evaluation of gene function prediction methods (CAFA2: [29]).

The CAFA2 bacteria proteins belong to 10 species (Table 1) and amount to 15,451. We added to this set other 2,187 bacteria proteins having at least one experimental GO annotation in the Uniprot knowledgebase/Swissprot (release: May 2013), but coming from organisms not considered in the CAFA2 challenge^a^, for a total of 17,638 bacteria proteins belonging to 301 different species.

**Table 1:** CAFA2 bacteria species and their proteins available in Swissprot (May 2013)

**ID.**	**Species**	**n. proteins**
83333	Escherichia *coli*	4431
224308	Bacillus *subtilis*	4188
99287	Salmonella *typhimurium*	1771
208964	Pseudomonas *aeruginosa*	1245
321314	Salmonella *entericacholeraesuis*	882
160488	Pseudomonas *putida*	693
223283	Pseudomonas *syringae*	675
85962	Helicobacter *pylori*	581
170187	Streptococcus *pneumoniae*	502
243273	Mycoplasma *genitalium*	483

The first column reports the SwissProt organism identifier, the last one the number of proteins.

**Figure 5:**
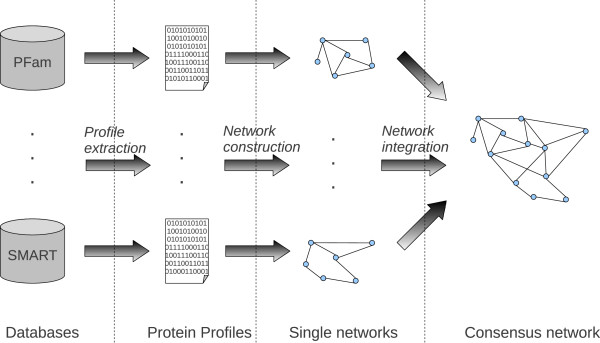
**Construction of bacteria net.** Data flows from different sources of information, construction of the data-type specific networks and networks integration.

Figure 5 sketches the main steps for the construction of the net of bacteria proteins. At first, we have collected data from the different databases reported in Table 2 to obtain different profiles for each protein. More precisely, each protein has been associated to a binary feature vector, representing a protein profile, whose elements are 1 when the protein is annotated for a specific feature (e.g. includes a specific domain, or a specific motif), or 0 otherwise (second phase in Figure 5). The protein profiles have then been used to construct a set of similarity networks (one for each data type) with edge scores based on the computation of the classical Jaccard similarity coefficient between each possible pair of protein profiles, thus obtaining 8 protein networks. Then we constructed two additional networks by computing the hierarchical Jaccard similarities between the Molecular Function (MF) and Cellular Component (CC) profiles associated to each protein and populated only with the experimentally supported GO annotations previously extracted from Swissprot (May 2013). The hierarchical Jaccard index is computed in the same way of the classical Jaccard, but the components of the vector (the GO terms) are weighted according to their distance from the leaves: GO terms corresponding to the leaves have weight *w*=1, those at distance *d*=1 weight *w*=1/2, and more in general nodes at distance *d* have weight $w=\frac{1}{d+1}$. In this way we put more emphasis on the most specific annotations, and two proteins annotated with the same more specific terms receive a similarity score larger than that obtained by two proteins annotated with less specific GO terms.

**Table 2:** Public databases exploited for the construction of protein profiles

**Database**	**Description**
Pfam [30]	Protein domain
Protein superfamilies [31]	Structural and functional annotations
PRINTS [32]	Motif fingerprints
PROSITE [33]	Protein domains and families
InterPro [34]	Integrated resource of protein families, domains and functional sites
EggNOG [35]	Evolutionary genealogy of genes: Non-supervised Orthologous Groups
SMART [36]	Simple Modular Architecture Research Tool (database annotations)
Swissprot	Manually curated keywords describing the function of the proteins
	at different degrees of abstraction

The 10 protein networks constructed according to the previously described steps have been integrated in an unique “consensus” network using the *Unweighted Average* (UA) network integration scheme [37]: the weight of each edge is computed by simply averaging across the available *n* networks, and “missing data”, i.e. pairs of vertices *i*,*j* not present in a given network, result in a weight *w*_*i* *j*_=0:

(5) $${\bar{w}}_{\text{ij}}=\frac{1}{n}\overset{n}{\underset{d=1}{\sum }}w_{\text{ij}}^{d}$$

where ${\bar{w}}_{\text{ij}}$ is the weight of the integrated network and $w_{\text{ij}}^{d}$ represents the weight associated to the edge (*i*,*j*) of the *d*^*t* *h*^ network (final phase of Figure 5).

As class labels for the proteins included in our integrated network we used the Gene Ontology Biological process (BP) experimental annotations extracted from Swissprot (May 2013). To ensure the availability of a reasonable amount of vertices from which to start the exploration of the direct and indirect neighborhood in the integrated protein network, we discarded all the GO BP classes with less than 20 annotated proteins, and this led to a final set of 381 GO terms with an amount of positives varying from 21 to 2,000 (Table 3).

**Table 3:** Summary of the distribution of the number of positives across the 381 GO BP classes involved in the functional labelling of the 17638 proteins comprised in the bacterial multi species protein network

**Min.**	**1st Qu.**	**Median**	**Mean**	**3rd Qu.**	**Max.**
21.0	31.0	53.0	135.4	131.0	2000.0

The performance of the considered methods have been quantified both in terms of area under the receiving operating curve (AUC) and precision at different recall levels in a standard 5 folds stratified cross validation scheme. We compared the execution times required for the completion of each ranking task in primary memory (on a machine with 16 Gb of RAM) and in secondary memory (on two machines with 16 and 4 Gb of RAM). The machine with 16 Gb of RAM has been equipped with an i7 Intel core processor with 8 cores at 2.80 GHz, while the 4 Gb machine with an Intel i7 core processor with 4 cores at 1.90 GHz (both have been equipped with off-the-shelf ATA hard disk drives). Both the machines run an Ubuntu 12.04 Linux operating system.

#### Eukarya-net

In order to test the ability of the proposed local methods to scale to large multi-species networks, we constructed a second network (hereafter referred to as *Eukarya-net*). Instead of considering different types of data, as we did in the construction of *Bacteria-net*, all the proteins interactions composing *Eukarya-net* were downloaded in precomputed form from the STRING protein-protein interactions database. STRING [38] is a collection of networks composed by real and predicted protein-protein interactions (based on genetic data, physical data and literature data) and aims at providing a global view of all the available interaction data, including lower-quality data and/or computational predictions for as many organisms as feasible [39]. Since version 7, STRING adopted a two-layered approach when accommodating fully sequenced genomes: important model organisms and those for which experimental data are available from the “core genomes”, while all the other genomes represent the “periphery” [40]. Starting from the STRING interaction data (version 9.05), we selected all the Eukaryotic species in the core region of STRING having 10,000 or more proteins. Our choice is motivated by the expected high quality of the interactions coming from the core region of STRING. The selected Eukaryotic species are listed in Table 4.

**Table 4:** Selected species from the core region of the STRING protein networks database

**NCBI taxon ID.**	**Species**	**n. proteins**
3218	Physcomitrella *patens*	10352
3702	Arabidopsis *thaliana*	23576
7227	Drosophila *melanogaster*	12845
7739	Branchiostoma *floridae*	16418
8364	Xenopus (Silurana) *tropicalis*	13678
9031	Gallus *gallus*	13119
9258	Ornithorhynchus *anatinus*	13333
9606	Homo *sapiens*	20140
9615	Canis lupus *familiaris*	16912
10090	Mus *musculus*	20023
13616	Monodelphis *domestica*	15409
39947	Oryza sativa *Japonica*	13330
69293	Gasterosteus *aculeatus*	13307

Each species is represented by at least 10000 proteins.

This network includes proteins coming from 2 invertebrates (a lancelet of the genus Branchiostoma and the fruit fly), 3 plants (*Arabidopsis thaliana*, the rice *Oryza sativa* and the moss *Physcomitrella patens*), and 8 vertebrates including a frog, the chicken and 6 mammals. The total number of proteins in *Eukarya-net* is 202,442. This basic version of *Eukarya-net* is obtained by extracting from STRING all the interactions occurring between proteins of the selected core species. This led to an initial collection of 25,132,538 interactions. A simple connected components analysis revealed that this network is composed by 666 connected components of which only 13 composed by more than 30 vertices (and corresponding to the biggest connected components of the networks associated to each species). This “big” network is thus a collection of the protein networks of the selected species. In order to find a way to “connect” the core components, we extracted all the clusters of orthologous genes from the STRING website according to the following steps: 

• the cluster of orthologs ID obtained by the STRING team using the eggNOG database (we considered only clusters of type NOG: non-supervised orthologous groups);

• the filtering of each NOG cluster in order to remove the proteins not coming from the selected core species. Note that some NOGs are composed by more than 2,000 proteins, but after our filtering procedure each selected NOG is composed by no more than 10 proteins.

After these steps, we selected all the NOGs in which the number of proteins equals the number of species (i.e. NOG composed by 10 proteins coming from 10 species, NOG composed by 9 proteins coming from 9 species, and so on). We finally constructed an enriched version of the basic *Eukarya-net* network simply by including in *Eukarya-net* all the possible edges linking the members of the selected set of NOGs. Following this strategy we obtained a network composed by 25,155,631 edges (network density: 0.000613). In order to verify the impact of the additional 23,093 NOGs based edges on the connectivity of *Eukarya-net*, we repeated the connected components analysis and we found that this “NOGs augmented” version of *Eukarya-net* is composed by 552 connected components of which two (190,755 nodes (94.22%) and 10,233 (5.05%)) account for more than 99% of the 202,442 proteins composing the network.

As class labels for the proteins included in *Eukarya-net* we used the GO annotations available in STRING (version 9.05). The STRING website provides flat text files containing a mapping from GO annotations to STRING proteins and a STRING internal confidence score for each GO annotation, ranging from 1 (low confidence) to 5 (high confidence). While extracting the GO labels we considered only the annotations with confidence score 5. We then filtered out all the GO terms associated with less than 20 and more than 100 proteins (473 GO terms). We finally randomly selected from this set 50 GO terms irrespective of their GO division (Molecular function, Biological process and Cellular component). We then repeated all the test performed on *Bacteria-net* on the bigger *Eukarya-net* network.

### Results and discussion

We compared the runtime required by main memory and secondary memory-based implementations (Section “Secondary memory-based computation”) of the *RW* algorithm described in Section “Local implementation of network-based algorithms”. Moreover, even if our main aim consists in showing that the combination of local implementation and secondary memory-based computation allows the analysis of big biological networks on small computers, we performed also a comparison of the performance achieved with single-species and multi-species networks of bacteria proteins to experimentally assess the impact of a multi-species approach to the prediction of protein functions.

#### Results with *bacteria-net*

Table 5 shows the average per term runtime required to complete a 5-fold cross-validation on the *Bacteria-net* (17,638 nodes/proteins and more than 7 millions of edges). We considered 381 GO BP terms characterized by more than 20 annotations and involving 301 species of bacteria. (see Section “Bacteria-net” for details). Results on the desktop computer (16 Gb RAM machine) show that the computational time required by the secondary memory based implementations, even if larger, is of the same order of magnitude of the time needed by the main-memory-based implementation. In particular, quite surprisingly, the empirical time complexity of the *GraphChi* implementation is very close to that of the the main-memory version. This fact can be partially explained by the very efficient secondary memory access of *GraphChi*, but above all by the characteristics of the main-memory implementation of the *RW* algorithm. Even if the efficient BLAS-based fortran subroutines for linear algebra are used for the classical stochastic matrix/probability vector product (eq. 1), the sparsity of the *Bacteria-net* network is not adequately exploited.

**Table 5:** **Empirical time complexity of the main and secondary memory-based implementations of network based algorithms for multi-species function prediction with the *Bacteria-net***

	**16 Gb RAM machine**			**4 Gb RAM machine**		
***Algorithm***	***Main mem.***	***Neo4j***	***GraphChi***	***Main mem.***	***Neo4j***	***GraphChi***
*RW - 1 step*	8.11s	27.92s	8.84s	–	208.27s	12.32s
*RW - 2 steps*	16.05s	54.36s	16.98s	–	408.57s	25.06s
*RW - 3 steps*	23.95s	81.18s	25.12s	–	621.92s	36.51s

The results of the main-memory algorithm with the notebook (4 Gb RAM machine) are not reported since on this task the main memory implementation of the algorithm fails, due to disk trashing, by which processor time is mainly used to continuously swap from main memory and the virtual memory on disk. On the contrary, the *GraphChi* implementation results only in a small increment of the computational time, mainly due to the larger time required to construct the shards when less RAM memory is available (Section “GraphChi: a disk-based system for graph processing”) and to the lower speed of the processor in the notebook machine.

Note that with the smaller machine the empirical computational time required by *Neo4j* increases of about one order of magnitude, while the *GraphChi* implementation introduces only a small increment of the required execution time (Table 5). This is particularly relevant when we consider the overall computational time required to predict the 381 GO terms: with the “small” machine *Neo4j* moves from about 3 hours to about one day with the 1-step *RW*, and from about 7 hours to almost 3 days with the 3-steps *RW*.

Even if the main aim of this work consists in showing that secondary-memory based technologies allow us to analyse large multi-species networks also with “relatively small” stand-alone computers, we report also the average AUC, and precision at 20 and 40% recall across the considered 381 GO BP terms. Table 6 shows that *RW* algorithms achieve reasonable results (AUC is always significantly larger than 0.5). In particular 1-step *RW* obtains the best results in terms of both AUC and P20R and P40R: on the average, the direct neighbours of each node seem to be the most informative.

**Table 6:** ***Bacteria-net*: average AUC, precision at 20% recall (P20R) and precision at 40% recall across 381 GO BP terms estimated through 5-fold cross-validation**

**Algorithm**	**AUC**	**P20R**	**P40R**
*RW - 1 step*	0.8744	0.2264	0.1673
*RW - 2 steps*	0.8590	0.1318	0.0893
*RW - 3 steps*	0.8419	0.1064	0.0713

#### Results with *Eukarya-net*

Table 7 summarizes the average per-term runtime required to complete a 5-fold cross validation with the *Eukarya-net* involving more than 200,000 proteins of 13 multi-cellular eukarya organisms (Section “Eukarya-net”). The spatial requirements induced by *Eukarya-net* prevents the application of the main memory implementation also with the 16 Gb RAM machine, while secondary memory-based implementations make this task feasible also with this large protein network.

**Table 7:** ***Eukarya-net*: Average per-term empirical time complexity between** ***Neo4j* and** ***GraphChi* implementations**

	**16 Gb RAM machine**		**4 Gb RAM machine**	
***Algorithm***	***Neo4j***	***GraphChi***	***Neo4j***	***GraphChi***
*RW - 1 step*	189.60s	20.44s	2520.00s	21.46s
*RW - 2 steps*	367.82s	31.68s	4919.35s	33.19s
*RW - 3 steps*	549.84s	45.73s	7333.10s	46.69s

It is worth noting that in this task involving a bigger net, the *GrapChi* implementation is significantly faster than the *Neo4j* implementation (Table 7). Moreover, the average computational time is in practice the same when the 4 Gb and the 16 Gb RAM machines run the *GrapChi* implementation of the *RW* algorithm, while we observe a relevant increment in computational time with *Neo4j*, as previously observed also with *Bacteria-net*.

The performance in terms of the average precision at fixed recall levels obtained in this test are relatively low, especially when compared with the high average AUC obtained with the *RW* at 1, 2 and 3 steps (Table 8). The observed relatively low precision can be explained by taking into account that it is more negatively affected by class unbalance and, in the *Eukarya-net* network task, the positives are at most 100 while the number of vertices in the network is 202,442 (i.e. the positives are less than 0.05% of the vertices at best). Note that in this case the 2-steps *RW* achieves the best AUC results: it is likely that these results could be due by the eggNOG orthology relationships added between the single-species disconnected components in *Eukarya-net* (Section “Eukarya-net”). Indeed in this way the annotations for a certain species can be propagated to other philogenetically related species by exploiting the orthology relationships.

**Table 8:** ***Eukarya-net*: average AUC, precision at 20% recall (P20R) and precision at 40% recall across 50 GO terms estimated through 5-fold cross-validation**

**Algorithm**	**AUC**	**P20R**	**P40R**
*RW - 1 step*	0.8601	0.1449	0.0943
*RW - 2 steps*	0.9667	0.1329	0.0929
*RW - 3 steps*	0.9598	0.0927	0.0785

#### Experimental comparison between multi-species and single-species approaches

In this section we provide an experimental comparison between multi-species and single-species approaches to *AFP*. We repeated the same *AFP* task performed with *Bacteria-net* but considering this time each species separately. More precisely, we constructed a separate net for each species of Bacteria, using exactly the same data we used for the multi-species net (Section “Bacteria-net”), and then we predicted the probabilities for each of the 381 GO terms considered in the multi-species task (Section “Results with *bacteria-net*”). Average per-species results show that the multi-species approach, by exploiting the multi-species network of proteins *Bacteria-net*, achieves better results in terms of both AUC, and precision at a fixed recall rate (Table 9), and the difference is statistically significant independently of the number of steps and the performance measure considered (Wilcoxon signed rank test, *α*=0.01).

**Table 9:** Comparison of the average AUC, precision at 20% recall (P20R) and precision at 40% recall between multi-species and single-species approaches with 301 species of bacteria

**Multi-species approach**			
**Algorithm**	**AUC**	**P20R**	**P40R**
*RW - 1 step*	0.8744	0.2264	0.1673
*RW - 2 steps*	0.8590	0.1318	0.0893
*RW - 3 steps*	0.8419	0.1064	0.0713
**Single-species approach**			
**Algorithm**	**AUC**	**P20R**	**P40R**
*RW - 1 step*	0.8263	0.1801	0.1176
*RW - 2 steps*	0.8146	0.1059	0.0647
*RW - 3 steps*	0.8179	0.1009	0.0563

These results can be explained, considering two characteristics of multi-species networks: 1) the number of nodes and the number of available annotated proteins; 2) the overall topology of the network.

Indeed in single-species nets either the reduced number of available proteins or the reduced number of annotated nodes can negatively affect the generalization capabilities achieved with random walks or any other learning algorithm, while in multi-species networks, by construction, more nodes and more annotated proteins from other species can be available.

Moreover in single-species networks usually the number of available functional connections (edges) between proteins can be reduced (for instance, since no sufficient data are available) and in many cases we may have highly disconnected networks, making very difficult the application of algorithms based on the propagation of the information between nodes. On the contrary, in the multi-species setting learning algorithms can enjoy a richer network topology by exploring connections not available in single-species nets: the evolutionary relationships between species assure that proteins not connected with other proteins of the same species, can in principle be connected with other homologous proteins in other species, thus enhancing the propagation of the information across the multi-species network.

Summarizing, our results show the feasibility of the “vertex-centric” algorithmic approach coupled with secondary memory-based technologies to process large multi-species protein networks with single off-the-shelf computers. Moreover, our preliminary experiments show that in perspective we can also improve performances by constructing large multi-species networks, and by integrating heterogeneous sources of biomolecular and evolutionary information.

## Conclusions

Our approach based on local implementations of network-based algorithms and on novel secondary memory-based technologies provides a solution to the large main memory requirements induced by large multi-species protein networks, thus making possible the analysis of big networks using off-the-shelf machines. Our results show that both graph DB technologies (i.e. *Neo4j*) and secondary memory based systems for graph processing (i.e. *GraphChi*) can be successfully applied to the analysis of large multi-species networks, even if the latter seems to be less sensitive to the amount of available primary memory, and more efficient for the implementation of network-based algorithms for AFP. The local implementation strategy can be applied to other network-based learning algorithms, ranging e.g. from simple guilt-by-association methods (that are inherently local) [41,42] to more complex label propagation methods [9,10], kernelized graph algorithms [25,43,44] and the recently proposed parametrized Hopfield networks [45], but in principle any algorithm, that can be expressed according to a “vertex-centric” programming model, can be adapted to this framework.

In perspective, by exploiting orthologous genes and multiple genomic sources, multi-species prediction can be applied to annotate poorly annotated species and discover new functions for uncharacterized genes in model organisms. Indeed our proposed approach allows computational biologists to experiment with large multi-species networks using their own notebooks, but in perspective applications to huge networks including e.g. the proteomes available in SwissProt/TrEmbl could be performed using well-equipped stand-alone machines.

Our framework could be also adapted and devised to other relevant computational biology scenarios characterized by the construction and processing of large networks, such as in the context of the “Network medicine” [46], or in drug discovery and repositioning problems [47].

## Availability of supporting data

The files containing the *Bacteria-net* and *Eukarya-net* along with the files containing the labels used in our experiments are available from GigaDB [48]http://dx.doi.org/10.5524/100090. The content and format of each file is described in readme files available at the aforementioned database.

## Endnote

^a^ For experimental annotation we considered all the available associations having GO evidence codes not included in the following list: IEA, ND, IC, NAS, TAS, ISS, ISO, ISA, ISM, IGC, IBA, IBD, IKR, IRD and RCA. A complete list of the GO evidence codes and their meanings is available at http://www.geneontology.org/GO.evidence.shtml.

## Abbreviations

AFP: Automated function prediction; BP: Biological process; CC: Cellular component; GO: Gene ontology; MF: Molecular function; RW: Random walk.

## Competing interests

The authors declare that they have no competing interests.

## Authors’ contributions

MM analyzed the technological items behind secondary memory based computation and implemented the *RW* algorithm using Neo4j; MR curated the biological items of multi-species AFP and implemented the same algorithm with GraphChi; GV wrote the draft (with the contribution of the other authors) and implemented the main-memory version of the algorithms. All authors read and approved the final manuscript.
